# Sensitivity Improvement of Multi-Slot Subwavelength Bragg Grating Refractive Index Sensors by Increasing the Waveguide Height or Suspending the Sensor

**DOI:** 10.3390/s22114136

**Published:** 2022-05-29

**Authors:** Siim Heinsalu, Katsuyuki Utaka

**Affiliations:** Faculty of Science and Engineering, Waseda University, 3-4-1 Okubo, Shinjuku, Tokyo 169-8050, Japan; utaka@waseda.jp

**Keywords:** bragg gratings, silicon optics, optical sensors, waveguide shape modification

## Abstract

We present two methods of improving wavelength sensitivity for multi-slot sub-wavelength Bragg grating (MS-SW BG) refractive index sensors. The sensor structure is designed to have high optical mode confinement in the gaps between the silicon pillars whereby the surrounding medium interaction is high, thus improving the sensitivity. Further sensitivity improvements are achieved by increasing the waveguide height or suspending the sensor. The second option, sensor suspension, additionally requires supporting modifications in which case various configurations are considered. After the optimization of the parameters the sensors were fabricated. For the case of a waveguide height increase to 500 nm, the sensitivity of 850 nm/RIU was obtained; for sensor suspension with fully etched holes, 922 nm/RIU; for the case of not fully etched holes, 1100 nm/RIU; with the sensor lengths of about 10 µm for all cases. These values show improvements by 16.5%, 25%, and 50.5%, respectively, compared to the previous result where the height was fixed to 340 nm.

## 1. Introduction

The main requirements for current sensing applications are high-speed and high precision for sensing any kind of substance in real time. These requirements can be satisfied by utilizing optical sensors [1,2]. Employing optical sensors has the additional advantages of a small size, a low cost of fabrication, mass production availability, robustness, and the possibility of lab-on-chip applications [3,4,5,6]. The potential for downscaling and mass production on a single platform is possible due to the high index–contrast ratio that allows propagating light to be trapped in relatively small waveguides on the order of a few hundred nanometers. When comparing various platforms, one of the best options would be silicon photonics on a silicon-on-insulator (SOI) platform with a standard waveguide (WG) height and a width of 220 nm × 400 nm, respectively [7]. Silicon photonics itself has been advancing rapidly in terms of both performance and capability. The reasons for this advancement of silicon photonics could be its compatibility with complementary metal-oxide semiconductor foundry processes, advances in its fabrication processes such as extreme UV lithography, and/or algorithmic methods for optimization such as inverse design, all of which reduce the fabrication cost and make it possible to miniaturize the optical devices [4,8,9].

Sensing with the optical devices as a biosensor is executed by utilizing a resonant device that can detect a tiny change in the surrounding medium in which this tiny change in its refractive index (RI) will cause a noticeable shift to the resonant peak in the simplistic approach. Various common resonant and interference-based devices have already been demonstrated to be effective for the sensing approach, such as micro ring resonators (MRR) [10,11], Mach–Zehnder interferometers (MZI) [12,13], photonic crystal cavities (PhC) [14,15], and Bragg gratings (BG) [16,17].

The sensing capabilities of the sensors can be categorized by the limitations we set. By seeking rather simple structures and resultantly easy fabrication with MZI sensors they have large footprints in the order of a few hundred µm. MRR sensors have high bending losses at smaller radiuses of about a few µm. These losses, of course, could be reduced with various modifications such as increasing the lateral index–contrast ratio or by changing the bend of the curvature [18,19]. An additional necessary step for practical sensors is optimization before fabrication by employing various simulation-tools [20]. To make this process fast and simple while having few parameters is necessary from viewpoint of time consumption and reproducibility. From the aspect of simple structures, optical fibre sensors with BGs have been widely used and in silicon photonics have proven to be available in quite compact sizes [21,22,23].

BGs with silicon photonics are a rather new type of sensor with the first experimental report in 2008 demonstrating a sensitivity of 33.6 nm/RIU with a BG length of 172 µm [24]. The low sensitivity and large length were due to the use of rib WGs with top etched BGs where the optical mode is mostly confined in the WG itself and the stop band (SB) depth was influenced from a BG depth. Improvements toward higher sensitivity are made by modifying the WG shape and optimizing its parameters so that optical mode will have increased interaction with the sensed medium.

For example, using rib WGs with side gratings can reduce the required etching steps to one. Slotted WGs with side gratings allow for more enhanced mode interaction with the sensed medium in TE mode. In addition, to enhance TM mode interaction, subwavelength WGs with BG condition can be used. The respective sensitivities for these structures of 59 nm/RIU, 340 nm/RIU, and 507 nm/RIU were demonstrated [25,26,27]. Further sensitivity improvement could be realized via the combination of the last two options, in which case it would have single multi-slot subwavelength (MS-SW) BGs, whereas with the standard WG height of 220 nm a sensitivity of 579 nm/RIU was initially demonstrated [28]. It should be noted that based on the periodic structure approach it can be also regarded as a PhC sensor [29]. Further sensitivity improvement was obtained with rigorous optimization, and it was suggested that for a WG height higher than the standard one a higher sensitivity of 730 nm/RIU could be expected with WG heights of 340 nm [30].

The same MS-SW BG as a basis will be applied in this article, with a further height increase up to 500 nm obtaining a sensitivity of 850 nm/RIU with a sensor length of only 8.6 µm. The secondary approach to sensitivity improvement is to introduce sensor suspension as demonstrated effectively with the PhCs, thus improving the sensitivity from 410 nm/RIU with a slotted PhC sensor [31] to 656 nm/RIU with a suspended slotted PhC sensor [32]. To have the MS-SW BG as a suspended sensor, suspension supporting modifications are necessary. The investigated sensors with modified structures are named based on these shapes: SAW BG, complex-SAW (CSAW) BG, and complex-WIRE (CWIRE) BG. Experimentally, the obtained best sensitivities were 913 nm/RIU for SAW BG and 922 nm/RIU or 1100 nm/RIU for CSAW BGs with sensor lengths of 11 µm, 10.8 µm, and 10.2 µm, respectively. As references, simpler sub-wavelength BG and suspended ladder BG structures were also studied.

For silicon BGs, the obtained sensitivity values were the highest reported so far. By avoiding the limitations of compact size at around 10 µm and by using a simple evaluation approach for the sensing peak, higher values were possible. For the structure using bimodal WGs of above 100 µm lengths, where both fundamental and secondary order modes interfere with each other at the end of the WG as with an MZI, a sensitivity of 2270 nm/RIU was obtained [33]. An MZI and MRR combination with a total length of above 5000 µm as a Vernier effect approach yielded a very high sensitivity of 21,500 nm/RIU [34]. Alternatively, by optimizing the MZI parameters, a particularly high sensitivity for the notch wavelength of 51,495 nm/RIU with arm lengths of 227.1 µm and 228.8 µm was reported [35].

In Section 2, the paper first briefly introduces the theory and the operation procedure. Section 3 is for simulation results, where firstly non-suspended and secondly suspended sensing approaches are compared using two-dimensional (2D) Finite Difference Eigenmode (FDE) with various transmission spectra of the BGs, followed by the optimization of the base MS-SW BG using 3D Finite Difference Time Domain (FDTD). Section 4 is the experimental section to explain the fabrication processes. Difficulties and precautions for fabrication are discussed in the Appendix A. In Section 5, the measured results of the fabricated sensors are shown to demonstrate their sensing capabilities. Section 6 is a short discussion, addressing the comparison between the measurements and the simulations, and the conclusion to summarize the paper is provided in Section 7.

## 2. Brief Theory and the Operation Procedure

**Figure 1:**
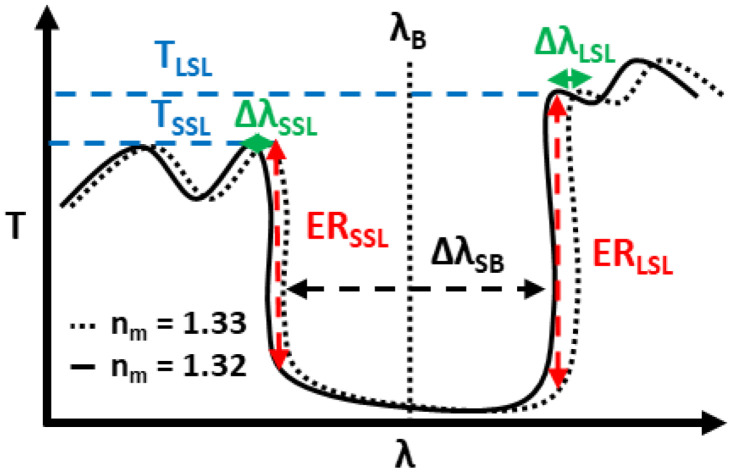
Schematic of transmission spectra for typical uniform MS-SW BG as a sensor.

A typical BG transmission spectral behavior regarding sensing performance is shown in Figure 1, where the reflected spectral range is called the stopband (SB) and its central wavelength corresponds to the Bragg wavelength (*λ_B_*) given as:

(1) $$m\lambda_{B}=2\Lambda n_{eff}\;$$

Λ is the BG period, *m* is the diffraction order, and *n_eff_* is the effective index of the BG WG. To have the largest diffraction efficiency *m* = 1 is taken. The spectral range of the SB (Δ*λ_SB_*) can be calculated from the coupled mode theory (CMT) with the following equations from reference [36]:

(2) $$\Delta \lambda_{SB}=\lambda_{B}^{2}/\pi n_{g}\sqrt{\kappa^{2}+{\left(\pi /\left(N\Lambda \right)\right)}^{2}},\;$$

(3) $$\kappa =2\Delta n_{eff}/\lambda_{B},\;$$

(4) $$n_{g}=n_{eff}-\lambda dn_{eff}/d\lambda ,\;$$

*N* is the period count, *κ* is the coupling coefficient, and *n_g_* is the group index. To have a compact sensor, the period of *N* = 20 in all the cases is adopted. To estimate approximate *n_eff_* and *n_g_* values, 2D FDE is applied on the cross-sections of the BG period each section by the following approach:

(5) $$n_{eff}=n_{1}\left(L_{1}/\Lambda \right)+n_{2}\left(L_{2}/\Lambda \right)+n_{3}\left(L_{3}/\Lambda \right)+\ldots ,\;$$

where *n*_1_ is *n_eff_* and *L*_1_ the length of the first BG section in the propagation axis. Knowing these values, Λ was selected to have the first sidelobes at the longer or shorter wavelength sides of the BG stopband with a wavelength of 1550 nm as a sensing peak. As CMT does not work well with high index perturbations additional correction is necessary. For this reason, 3D FDTD was employed to adjust Λ to have a sensing peak at 1550 nm.

To estimate the performance of the BG device as a sensor, the refractive indexes of the surrounding media *n_m_* are changed assuming various liquids such as pure and sugar-dissolved waters with *n_m_* of 1.32 and 1.33, respectively, at 1550 nm wavelength. By this medium refractive index change (Δ*n_m_*), the observed BG spectra will shift by a certain wavelength amount (Δ*λ*). For the uniform BGs, the Δ*λ* will be dependent upon which sidelobe peaks of the stopband are chosen, the shorter-wavelength sidelobe (SSL) or longer-one sidelobe (LSL). Not only the wavelength shift Δ*λ* but also the transmission (T) change, that is, the extinction ratios (ER) at both stopband ends, is effective to evaluate sensitivities depending on the specific sensor structures such as a larger height WG one, as shown in Figure 1. In the case of the base MS-SW BG configuration, it was previously observed that the LSL side was more effective [30]. However, the study of a suspended MS-SW BG will show that the SSL is more effective.

For the sensors studied here, the output lights include various effective sensing parameters such as the wavelength change depicted as the wavelength sensitivity (*S_*λ*_*) given by [30]:

(6) $$S_{\lambda }=\Delta \lambda /\Delta n_{m},$$

where Δ*n_m_* is the refractive index change of the surrounding medium. This parameter will be one of the main parameters of interest to be improved. The sensing scheme of the wavelength sensitivity needs either of these measurement sets: (a) a broadband light source and an optical spectral analyser, or (b) a tuneable light source and a photodiode to measure spectral behaviours. These measurement sets may be rather expensive but would provide higher accuracy due to the high-resolution performance of a tuneable light source and an optical spectral analyser.

The other important sensing parameter is the transmittivity change measured by the output power for a fixed wavelength as the extinction ratio (ER), and this is the amplitude or the power sensitivity given by the following [37]:

(7) $$\;S_{a}=\frac{1}{T_{\max}}\frac{\partial T}{\partial n_{m}}$$

where 1/*T*_max_ is for the normalization and *∂T*/*∂n_m_* the slope of the sensing peak by the medium refractive index change. The sensing measurement scheme of amplitude sensitivity can be configured by cheaper equipment sets such as those using only a single-wavelength light source and a photodiode, but the single-wavelength of the light source should be critically adjusted at the appropriate one.

Aside from the equipment comparison, the two measurement schemes, the use of either wavelength sensitivity or amplitude sensitivity, each have pros and cons from the viewpoint of their performances. For the wavelength sensitivity sensor, it is rather easy to recognize the specific wavelength at the sidelobe peaks to be measured by using a high-resolution optical spectral analyzer. These sharp sidelobe peaks of the MS SW BG sensors are a result of the slow light effect of the high-index modulation grating structures. However, the wavelength sensitivity sensor basically needs some measurement time to scan the measurement wavelength range, which is not suitable for real time measurement. On the other hand, the amplitude sensitivity sensor has the advantage of fast real time measurement since it only detects a transmitted light power, but the sensitivity is critically dependent on the slope steepness of the transmission spectra around the sidelobe peak, which is easily degraded by transmission loss of the device.

It should be noted that the MS SW BG sensors utilize their intrinsic spectral features with sharp sidelobe peaks, and these wavelength characteristics are required to respond only to the refractive index change of the surrounding medium, not, for example, by the temperature change which changes the refractive indexes of the silicon waveguide as well as the sensed medium. An allowable temperature change will be discussed later.

## 3. Simulation Results

### 3.1. Comparisson of Sensing for Sunspended and Non-Suspended Sensors

To simplify the discovery of the optimal sensing parameters, the sidelobe wavelength shifts (Δ*λ*_SSL_ or Δ*λ*_LSL_) of the Bragg grating are considered as comparable to the medium RI change Δ*n_eff_*, as:

(8) $$\Delta \lambda_{SSL}\sim \Delta \lambda_{LSL}\sim \Delta n_{eff}$$

**Figure 2:**
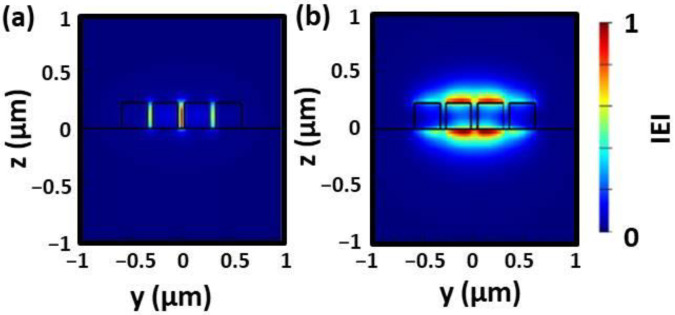
Optical intensity for Si MS-WG for (**a**) TE and (**b**) TM modes with SC = 3, W_s_ = 60 nm, W_t_ = 1100 nm, and H =220 nm.

Before taking this approach, it is necessary to analyze the propagating modes in MS-WG. The simulated results of the optical intensities indicate that the mode confinement for the fundamental TE mode is in the slot regions and for the fundamental TM mode in the top and bottom regions of the WGs, as shown in Figure 2. From these results, it is evident that the fundamental TE mode is interacting more with the surrounding medium, and the TE modes will be used herein.

Regarding the optimal SC value as found previously with SC > 2, we have approximately equal maximal sensitivity, but the optimal region tends to be smaller for other parameters with larger SC [30]. To avoid additional limitations in fabrication, for which the corners of the pillars would become round and the structure complex, an SC of 3 should be adopted.

**Figure 3:**
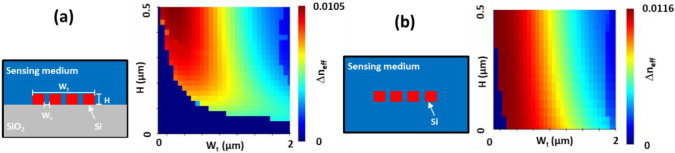
Mapping of Δ*n_eff_* for (**a**) non-suspended and (**b**) suspended Si MS-WGs depending on W_t_ and H with W_s_ = 60 nm and SC = 3.

Mapping Δ*n_eff_* for MS-WG as a function of height (H) and total width (W_t_) with a slot size (W_s_) of 60 nm shows that most effective sensor structures have a large WG height for the non-suspended WG, as seen in Figure 3a. While even H > 500 nm might increase the sensing effectiveness, it is easier to consider the usage of commercial chips with a standard WG height of H = 220 nm. As a second option, the suspended MS-WG was studied, as shown in Figure 3b. In this case the optimal structures for the higher sensitivities are for small MS-WG widths W_t_ of less than 500 nm regardless of WG heights. While smaller W_t_ values will provide higher sensitivity, the *n_eff_* will also gradually decrease and in association increase the transmission loss (TL) due to possible increased scattering, affecting and decreasing the sensing peak sharpness. If the *n_eff_* becomes smaller than *n_m_* it is categorized as a leaky mode.

### 3.2. Various Configurations for Sensing

**Figure 4:**
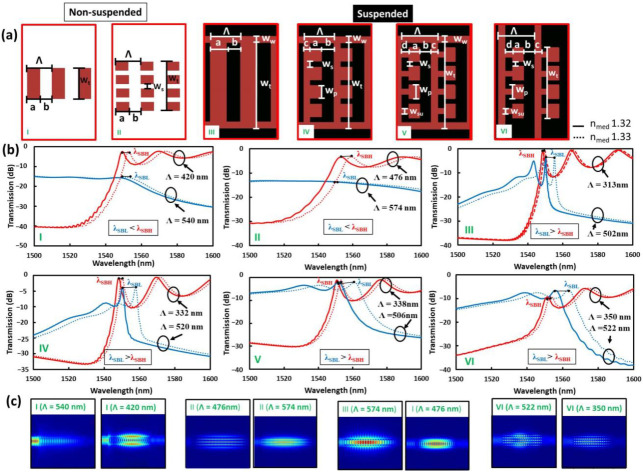
(**a**) Top views of the various BG RI sensors: I—SW BG, II—MS-SW BG, III- suspended ladder BG, IV– SAW BG, V—CSAW BG, and VI—CWIRE BG. (**b**) The respective transmission spectra with shorter (in blue) and longer (in red) wavelength-sides of the stopband at a fixed wavelength of 1550 nm fitted by Λ values. Parameters used are as follows: H = 220 nm (I, III–VI), 500 nm (II); W_t_ = 500 nm (I), 980 nm (II, VI), 600 nm (III), 1300 nm (III–V); W_s_ = 60 nm (II, IV-VI); W_su_ = 60 nm (II, V, VI); W_w_ = 100 nm (III–V); d = 100 nm (IV–VI); c = 100 nm (V, VI); the duty ratios for all of the cases is 0.5. (**c**) E-field top views for the various BGs.

To clarify the optimal structures and sensing parameters, 3D FDTD simulations with six configurations were investigated where two were for the non-suspended cases and four for the suspended cases, as shown in Figure 4.

For the non-suspended cases, the simple SW BG and the MS-SW BG structures are schematically shown in Figure 4a-I and II, respectively. The fundamental structural parameters used were common, with a WG height (H) of 500 nm and a total WG width (W_t_) of 500 nm for the SW BG, and a W_t_ of 980 nm with slot sizes (W_s_) of 60 nm for the MS-SW BG and the duty ratios of 0.5 with the required Λ calculated by the procedure mentioned in the previous chapter. By comparing the transmission spectra at SSL and LSL wavelengths for both structures, the LSL for the MS-SW BG was more efficient for higher wavelength sensitivity. This is firstly because it has additional slot regions and secondly due to it having more mode confinement in the surrounding regions outside of the Si waveguides for the longer wavelength side of the stopband, both enhancing the interactions between the propagating modes and the surrounding medium. On the other hand, for the SSL, wavelengths tend to suffer from scattering loss and resultantly have a decreased sharpness. These behaviors can also be observed with the E-field profiles in Figure 4c. As for the suspended cases, suspensions supporting the SW BG and MS-SW BG structures are required. The SW BG structure needs only side walls for suspension, as shown in Figure 4a-III, and it was named the suspended ladder BG in this instance. In the case of the MS-SW BGs, they need additional supporting structures such as beams crossing between the side walls to support each periodic block structure. As the periodic block structure is similar to backsaws, as in Figure 4a-IV, it was named SAW BG. In addition, when SAW BGs have bottlenecks to the crossbeams, as in Figure 4a-V, this configuration is called complex SAW (CSAW) BG.

Lastly, as another option for suspension, when long silicon wires are used as cross beams to support periodic blocks, as in Figure 4a-VI, named complex WIRE (CWIRE) BG.

The structural parameters of the various types of sensor used in the simulation, of which the respective notations are denoted in Figure 4a, are as follows: WG height H = 220 nm (for the types I, III–VI) and 500 nm (II); W_t_ = 500 nm (I), 980 nm (II, VI), 600 nm (III) and 1300 nm (III–V); W_s_ = 60 nm (II, IV–VI); W_su_ = 60 nm (II, V, VI); W_w_ = 100 nm (III–V); d = 100 nm (IV–VI); c = 100 nm (V, VI); duty ratios are 0.5 for all of these types.

It is interesting to note that the suspension-type sensors display both higher wavelength and amplitude sensitivities for the SSL wavelengths drawn in blue lines compared to those at the LSL one drawn in red lines, as is seen from Figure 4b-III to VI. By comparing all the cases, the sensitivity effectiveness from the highest to the lowest is CSAW > CWIRE > SAW > suspended ladder BG. This is due to greater expansion of the optical mode fields into the surrounding spaces below and above Si WGs as a result of the introduction of the open spaces below Si WGs.

### 3.3. MS-SW BG Sensing Improvement by WG Height Increase 

To optimize the MS-SW BG structures for higher sensitivity, the dependence on WG height H and additionally on pillar shapes was also analyzed. As for the pillar shapes, ellipsoid, rhombus, and rectangle were considered. While there might also be a possibility for the fabricated pillar shape to change to a rectangular frustum due to side etch issues in fabrication. This can be avoided by increasing the metal mask thickness for durability during the etching, as noted in the Appendix A.

**Figure 5:**
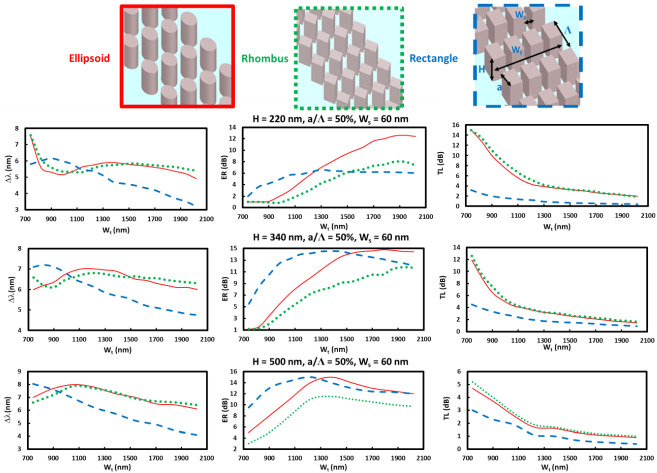
Sensing characteristics of MS-SW BG RI sensors for three pillar configurations schematically shown in the top Δ*λ*; ER and TL dependent on W_t_ at height of H = 220 nm, 340 nm, and H = 500 nm with a/Λ = 0.5 and W_s_ = 60 nm, where Λ is fitted to have sensing peak at 1550 nm.

The sensing parameters Δ*λ*, ER, and TL as a function of total WG width W_t_ for various WG heights are shown in Figure 5, with red, green, and blue lines corresponding to ellipsoidal, rhombus, and rectangular pillar structures, at various WG heights. 

One can see that the rectangular pillar tends to exhibit higher sensitivities in Δ*λ* and ER for narrower W_t_ and a larger WG height H than those of the other two pillar structures. This tendency seems to be the result of the lower material loss for the rectangular pillar, as shown in the TL characteristics of Figure 5. The optimal W_t_ is smaller for the rectangular pillar than that of the ellipsoidal and rhombus pillars and may be due to the larger volume ratio in the periodic unit volume, and this structural uniformity may reduce the loss. The reason why the larger H is better for the higher sensitivities may be due to the presence of more optical fields in the surrounding spaces between the pillars. Ellipsoidal and rhombus shapes may appear via deformation of the rectangular shape in the fabrication process. Depending on the fabrication issue, the optimal size conditions must be considered.

### 3.4. MS-SW BG Sensing Improvement by Suspension 

**Figure 6:**
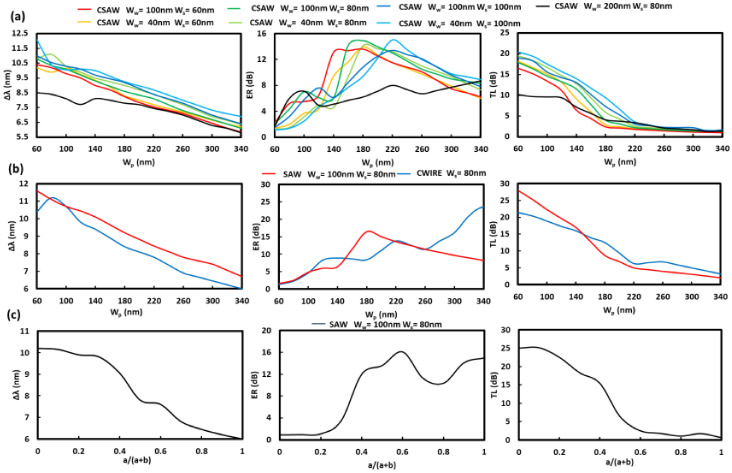
SAW, CSAW, and CWIRE BG RI sensors. Δ*λ*, ER and TL dependence on (**a**,**b**) W_p_ with various W_s_ and W_w_ values with constant values being W_su_ = 60 nm and c = d = 100 nm or (**c**) a/(a + b) for SAW BG RI sensor and (**b**) H = 500 nm. With Λ fitted to have sensing peak at 1550 nm.

Next, the structural optimizations for the suspended structures such as SAW, CSAW, and CWIRE BGs were carried out. First, the sensing characteristics of CSAW BG with a constant H of 220 nm for various combinations with wall widths W_w_ from 40 nm to 100 nm and slot widths W_s_ from 60 nm to 100 nm as a function of pillar widths (W_p_) were analyzed, as shown in Figure 6a. The wavelength sensitivities Δ*λ* linearly decrease with an increase of W_p_ similar to the tendency for an increase of W_t_ shown in Figure 5. For the structures with narrower walls W_w_ and larger slot size W_s_, the maximum Δ*λ* of 11.7 nm was expected at the cost of low ER and large TL. The low ER is due to the sensing peak widening and high TL due to the low mode confinement. Further, the behavior of Δ*λ* at small W_p_ seems to have higher values indicating that it corresponds to an optimal region. Optimal structures for higher ER seem to have two regions, in which the first is at a smaller peak at around W_p_ of 100 nm and the second at a more enhanced region at around W_p_ of 170 nm to 220 nm, depending on the corresponding modes and related stopband edges with their different steepnesses.

The second set of simulations are for the SAW BG with W_w_ of 100 nm and W_s_ of 80 nm, and the CWIRE BG with W_s_ of 80 nm for all the structures, and the results are shown in Figure 6b. The behavior of the sensing performances was fairly similar to the previous cases for CSAW BG.

The third set of simulations are for SAW BGs with similar W_w_ of 100 nm and W_s_ of 80 nm, however, as a function of the duty ratio defined as (a/(a + b)), shown in Figure 6c. The results are similar when considering W_p_ but the maximum Δ*λ* is smaller, and for ER the first optimal region is missing with the third optimal region being more prominent.

**Figure 7:**
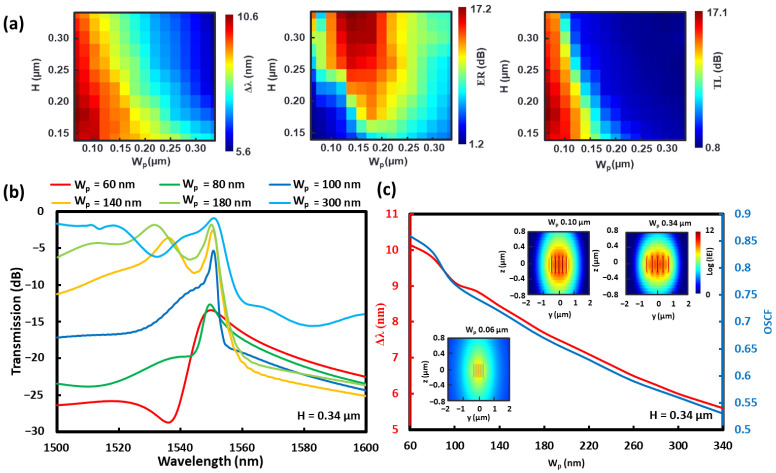
(**a**) Mapping of characteristics of CSAW BG RI sensor, Δ*λ*, ER and TL, dependent on H and W_p_ with other parameters as W_s_ = W_su_ = 60 nm, W_w_ = d = c = 100 nm, and N = 20. (**b**) Simulated transmission spectra for various W_p_ values with H = 340 nm and (**c**) simulated OSCF and dependent on W_p_. Insets show electric field profiles in logarithmic scale at 10th pillar area cross-sections.

As with the suspended MS-WG in Figure 3b, the sensing characteristics mappings for CSAW BG as a function of H from 140 nm to 340 nm, Wp from 60 nm to 340 nm, W_s_ = 60 nm, W_w_ = 100 nm, and the supporting blocks of 100 nm × 60 nm are shown in Figure 7a. As expected, the Δ*λ* mapping behavior is similar to the Δ*n_eff_* profile of Figure 3b. However, the large Δ*λ* dependence on H is more evident, which is due to the fact that the leaky modes were also affected for CSAW BG. In the case of ER mapping, two peak ER regions were observed. The first small region at W_p_ less than 80 nm and H ≥ 300 nm corresponds to the case where SSL slope orientation is flipped to the opposite direction as is indicated by the red and green lines in Figure 7b. With a further increase in W_p_, the slope is switched typical orientation. With this slope orientation the optimal region is larger with W_p_ from 100 nm to 180 nm and H ≥ 240 nm. For the CSAW BG case, the optical space confinement factor (OSCF), which is defined as the optical power ratio of the surrounding space to that of the total one [30], was also calculated as a measure of the RI sensitivities, as in Figure 7c. The results indicate that OSCF behavior is almost coincident with that of Δ*λ*, which means that the wavelength shift caused by the change of RI is enhanced by the greater amount of optical fields in the surrounding region, that is, the larger OSCF. However, it should be mentioned that a larger OSCF leads to greater transmission loss, as is clear in Figure 6, meaning that the structures with large OSCF tend to have larger TL. On the other hand, for the structures with lower TL, their amplitude sensitivities become greater, which indicates the opposite designs of the optimal sensor structures for high wavelength sensitivity with a large OSCF and the high amplitude ones with a small OSCF.

## 4. Fabrication

**Figure 8:**
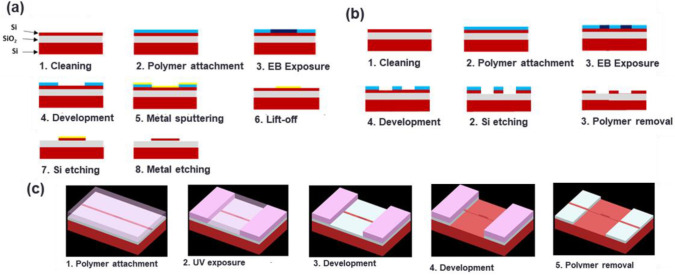
Sensor fabrication procedure steps (**a**) Lift-off method for WG formation, (**b**) inverse exposure method for WG formation, and (**c**) sensing region suspension.

The Si MS-SW BG sensors were fabricated on SOI substrates with two major steps. The first step was the WG formation employing (1) electron beam lithography (EBL), electron beam deposition (EBD), and inductively coupled plasma reactive ion etching (ICP-RIE) or (2) EBL and ICP-RIE, corresponding to Figure 8a,b, respectively. The second step was to form the suspension structure, if necessary, by employing ultra-violet lithography (UVL) and wet etching of the SiO_2_ under-cladding buried oxide (BOX) layer corresponding to Figure 8c. The SOI wafers that were used had either 500 nm or 220 nm-thick silicon and 2 µm-thick BOX layers. 

The first method of WG formation consisted of eight steps. First, the SOI substrates were cleaned by submerging them in acetone, isopropanol, and NMD-3% for 5, 10, and 3 min, respectively, and finally cleaned with pure water. Second, an EBL resist ARP-6200.09 was spin-coated, and then samples were pre-baked for 3 min at 180 °C. Third, EBL (ELS-7700 W, Elionix, Tokyo, Japan) was carried out at an acceleration energy of 75 keV for a field size of 150 µm^2^. Fourth, the exposed samples were developed with ZEP-N50 and rinsed with ZMD-B for 30 and 60 s, respectively. Fifth, 40 nm-thick nickel (Ni) film was deposited with EBD (EBX-6B, ULVAC, Kanagawa, Japan). Sixth, unwanted Ni was removed by lift-off step with NMD-3% at 130 °C for 30 min. Seventh, etching of 220 or 500 nm-thick silicon waveguide was performed by ICP-RIE (RIE-200iP, Samco, Kyoto, Japan) with gases of CF_4_+Ar in 8:2 mixture at a pressure of 0.1 Pa with silicon etch rate of about 98 nm/min. Finally, samples were submerged in chromium etchant at 130 °C for 20 min to remove Ni film, followed by final cleaning with pure water. 

The second method of WG formation consisted of six steps. The first four steps were similar to the previous method except that the negative EBL resist was used by inverting the patterns, and the thickness of the resist was around 6x thicker (480 nm compared to 80 nm). The large EBL resist thickness was necessary for the fifth step where the protective mask for ICP-RIE was the polymer itself. The etching gas that was used was replaced with SF_6_ with a silicon etching rate of 135 nm/min, and the adoption of thicker EBL resist was due to the high etching rate by SF_6_, of about 350 nm/min compared to 200 nm/min for the previous gas. Finally, in the sixth step, the EBL resist was removed by NMD-3% at 130 °C for 30 min.

Based on the fabrication procedure differences, the methods herein are named lift-off method for the first method and inverse exposure method for the second one. The difference in the performance was that the lift-off method can form patterns with smaller sizes; however, the success yield seems smaller due to the lift-off step’s difficulty. To achieve a higher success yield, the inverse exposure method was used at the cost of having to use a larger exposure area that increased the fabrication cost. Additionally, this method limits the WG height due to the smaller etching resistance of EBL resist. To avoid this limitation, metal-organic EBL resists are an option [38]. The exposure power that was used in both methods was 155 µC/cm^2^ at which point the differences of the actual size after etching compared to the design for line patterns were around +20 nm and −50 nm for the lift-off and the inverse exposure methods, respectively.

The fabrication process for the waveguide suspension consisted of five steps. First, AZ-5214 ultraviolet (UV) photoresist was deposited by spin coating followed by pre-baking on a hot plate at 100 °C for 1 min. Second, UV exposure of the suspension formation for about 8 s was carried out. Third, it was developed by NMD-3% for 45 s. Fourth, the BOX layer was etched off by buffered hydrofluoride acid (BHF) at 45 °C for 10 min for the lift-off method and 25 min for the inverse exposure method

## 5. Experimental Results

**Figure 9:**
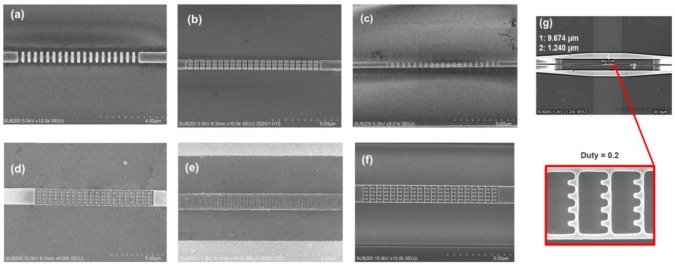
SEM images of fabricated BG RI sensors: (**a**) SW, (**b**) MS-SW, (**c**) suspended ladder, (**d**) CSAW formed by lift-off method, (**e**) CSAW formed by inverse exposure with not-fully etched or (**f**) with fully etched, and (**g**) SAW at duty of 0.2.

The SEM top-view images of the fabricated BG RI sensors are shown in Figure 9. The measurement setup and procedure were similar to those in the reference [30]. Single mode fibers were placed at the appropriate positions onto the input/output TE mode grating couplers. The fibers were connected to a tunable laser light source (TSL210, Santec, Aichi, Japan) with wavelength spans of 1500–1580 nm or 1530–1610 nm and an optical light power meter (Yokogawa, AQ2200-211 sensor module). For the measurements, the sensors were fully covered by a sample liquid medium whose refractive index change was sensed. First, the sample medium was pure water with an RI of 1.3164, and for the secondary measurements water with 5% sugar weight by concentration, glycerin with 10% weight concentration, or IPA, corresponding to the RIs of 1.327, 1.3302, and 1.3661, respectively, were used [39]. To avoid temperature influences on the performance as much as possible, all measurements were done at room temperature (RT) of 21 °C, and the sensors and the sensed mediums were kept for one hour in the same room before measurement for them to reach the same temperature. For each of the BG sensor types, seven different Λ valued samples were fabricated with the middle Λ value being equal to the calculated ones and others with +/− 10 nm increments. The adoption of different Λ values was to take into account possible deviations from the designed ones by side etching, corner rounding, and size differences. To obtain spectra of the sensors reference patterns were also fabricated with straight 500 nm wide WGs in the suspension areas.

**Figure 10:**
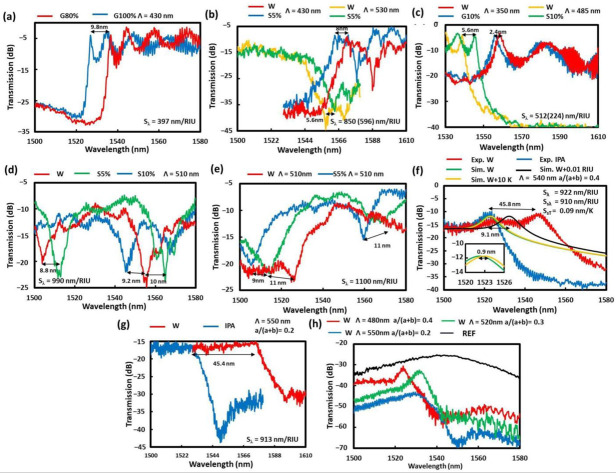
Measured transmission spectra for BG RI sensors with different surrounding medium liquids for (**a**) SW, (**b**) MS-SW, (**c**) suspended ladder, (**d**) CSAW by the lift-off method, (**e**) CSAW by the EBL inverse exposure with not fully etched holes, (**f**) with fully etched holes, and (**g**) SAW. (**h**) Measured SAW spectra for various duty ratios (a/(a + b)) in W medium. The surrounding media were W for water, Sx% (Gx%) for water with x weight concentration sugar (glycerol), and IPA for isopropyl alcohol. In (**f**) for CSAW, the simulated spectra are drawn as references for a sensing liquid with RI of pure water (W) at room temperature (RT), RI of W + 0.01, RIU at RT, and RI of W at RT + 10 K by green, black, and yellow lines, respectively.

The measurement results are shown in Figure 10 for the various sensor structures as indicated. The structural sizes for (a) non-suspended SW BG were W_t_ = 500 nm, H = 220 nm, duty = 0.4, and Λ = 430 nm, and the wavelength sensitivity of S_*λ*_ = 397 nm/RIU was obtained with TL of 4.8 dB. Those for (b) MS-SW BG were: W_t_ = 780 nm, W_s_ = 75 nm, H = 500 nm, duty = 0.7, and Λ = 430 nm for LSL or Λ = 530 nm for SSL, obtaining an S_*λ*_ of 850 nm/RIU with TL of 5.5 dB and 591 nm/RIU with TL of 20 dB, respectively.

The various suspended sensors in Figure 9 and Figure 10 correspond to (c) suspended ladder BG, (d) CSAW BG via the lift-off method, (e) CSAW BG via the inverse exposure method with non-fully etched holes, (f) CSAW BG via the inverse exposure method with fully etched holes, and (g) SAW BG via the inverse exposure method. As in Figure 10c, the best wavelength sensitivity for the suspended ladder BG sensor was obtained for the parameters of W_t_ = 700 nm, W_w_ = 100 nm, H = 220 nm, duty = 0.5, and Λ = 350 nm for LSL or Λ = 485 nm for SSL, obtaining S_*λ*_ values of 224 nm/RIU at TL of 5.1 dB for LSL and 512 nm/RIU at 4.7 dB for SSL. It was confirmed that LSL was more effective for the non-suspended cases and oppositely SSL for suspended cases. For Figure 10d, the best results of the CSAW BG by the lift-off method were attained for the parameters W_t_ = 1600 nm, W_w_ = 60 nm, W_s_= 140 nm, H = 220 nm, duty = 0.5, and Λ = 510 nm, obtaining S_*λ*_ of 990 nm/RIU. The wide W_t_ and W_s_ here were due to difficulties in the lift-off by using a thin Ni mask of 40 nm at the cost of avoiding the side etching and rounding issues. The problem of not being able to distinguish SB clearly could be due to two reasons: first, the W_s_ was too large, and second, a few pillars were missing. As in Figure 10e, the CSAW BG by inverse exposure with non-fully etched holes corresponds to a sample whereby using near 220 nm etching, a lag effect typically for GCs with circular hole patterns was observed [40]. The sensor parameters for the best sensitivity here were W_t_ = 1240 nm, W_w_ = 100 nm, W_s_ = 80 nm, H = 220 nm, duty = 0.5, etch depth around 190 nm, and Λ = 510 nm, obtaining an S_*λ*_ of 1100 nm/RIU. 

To compare the simulation reliability for the previous case, the fully etched CSAW BG, three simulation results were included in Figure 10f as sensed liquids with an RI of pure water (W) at RT, RI of W + 0.01 RIU at RT, and RI of W at RT + 10 K. Here, to include temperature effects, the thermo-optic coefficients for silicon and water 1.86 × 10^−4^ RIU/K and −1 × 10^−4^ RIU/K [40,41] were used. By comparison, the LSL location fits quite well with the experiment; however, the SB depth is much lower. By increasing the RI, the simulator wavelength sensitivity (S_λs_) was 910 nm/RIU. This slightly lower value can be explained by the possible temperature increase of around 1 K since the obtained simulator temperature sensitivity (S_sT_) was 0.09 nm/K. This is a value comparable to the previously reported temperature RI SOI sensors [42].

Finally, as in Figure 10f, in the third CSAW BG by inverse exposure, with an etch depth increased to 230 nm and slightly increased development time, the SBs were clearly visible. However, due to fabrication differences the sensor with the same parameters as the previous case had an S_*λ*_ of only 730 nm/RIU. The lower value might be due to not small deviations from the designed parameters. To increase the S_*λ*_, the value duty was decreased to 0.4 obtaining S_*λ*_ of 922 nm/RIU at TL of 11.8 dB.

To simplify the fabrication and still have such a high S_*λ*_ the next option was SAW BG by inverse exposure, as in Figure 10g, with a similarly reduced duty value. The parameters for the best S_*λ*_ were W_t_ = 1080 nm, W_w_ = 80 nm, W_s_ = 120 nm, H = 220 nm, and duty = 0.2, obtaining an S_*λ*_ of 913 nm/RIU at TL of 15.5 dB. A comparison of the various duty ratios, 0.4, 0.3, and 0.2, along with the output spectrum from the device without any sensor in the suspension as a reference (REF) is shown in Figure 10h. From these results, it was found that the TLs at the sensing peaks increased and their peaks widened with a decrease of the duty ratios.

## 6. Discussion

**Figure 11:**
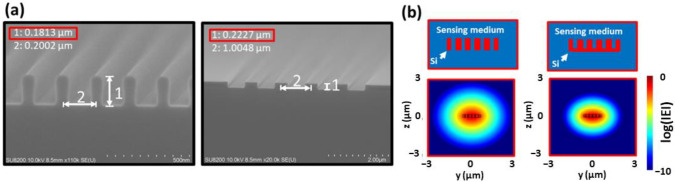
(**a**) Observed lag effect on a silicon wafer samples with grating periods of 200 nm (**left**) and 1 µm (**right**) with duty of 0.5 by inverse exposure method. (**b**) Schematic cross-sections of the MS WG and respective E-field profiles with fully etched (**left**) and not fully etched holes (**right**), with design parameters the same as in Figure 10e.

In Figure 5 and Figure 7, the effects of the shape deformation of the pillars and periodic structures of the sensors were discussed to give the deviation of the optimal structures designed for high performances, and the experimentally obtained sensitivity that was higher than the simulated one was explained from the shape deformation from rectangular to round shapes after the fabrication leading to the effective increase of OSCF. This deformation possibly originated from EB lithography, such as lift-off or inverse exposure techniques, and was related to the SW BG formation process by etching to silicon waveguides with EB resist or metal masks. The EB resist patterns that were formed critically depended on EB dose and development time, which is known as the so-called proximity effect where for smaller exposed areas actual exposure tends to be weaker [43]. To avoid this, a +5 s time adjustment in ZED-50 development and a +10 nm additional etching time were taken into account. During the etching process, there was a lag effect for smaller exposed areas whereby the WG inner etching region was not fully etched. The example of this lag effect is shown in Figure 11a, which shows the etched silicon gratings for two different periods of 200 nm and 1000 nm and with almost the same etching depth for around 220 nm. The actual etched depth was smaller for the smaller period grating, resulting in insufficient etching in some cases. The simulated TE mode profiles are shown in Figure 11b for the non-fully etched and the fully etched multi-slot structures in this case, indicating the larger TE mode confinement in the slot regions for the non-fully etched case. This lag effect also affects the grating duty ration, and these results suggest that the fabrication process should be undertaken with great attention paid to the designed structures in MS-SW BG sensors.

Finally, the temperature’s effect on the sensor performance is discussed phenomenologically. The sensors investigated in this study are basically utilizing periodic structures, and the sensing wavelengths are set at the sidelobes of the SB whose center wavelength is the Bragg wavelength of λ_B_ = 2*n_eff_*Λ, where Λ is the BG period and *n_eff_* is the effective index of the periodic structure determined by RI averaging of the WG structure. For the light propagation in the periodic structure, especially for the wavelength around the stopband, its phase is quite dispersive, and its propagating velocity is delayed by a factor of the ratio n_eff_/n_g_, where n_g_ is a group index and is larger than *n_eff_*. This is the so-called slow light effect and it plays the role of sensitivity enhancement and resultantly the shortening of the sensor length as well as the effect of the mode profile modification evaluated by OSCF. Here, the relative temperature coefficient of the wavelength sensitivity S_*λ*_ can be derived as (1/S_*λ*_) (ΔS_*λ*_/ΔT) = |(1/*n_eff_)*(Δ*n_eff_*/ΔT)|. The last term Δ*n_eff_/*ΔT is related to the thermo-optic (TO) effects of the waveguide mediums including silicon WG itself and a surrounding medium, where the TO coefficients of silicon and water were set as 1.86 × 10^−4^ RIU/K and −1 × 10^−4^ RIU/K, respectively [40,41]. The temperature sensitivity was about 0.09 nm/K, as shown in Figure 10f, and the relative temperature coefficient is in the order of less than × 10^−4^. It is noted that the temperature effect may be compensated by the opposite sign of the TO coefficients of silicon and the conventional liquids to be sensed.

In addition to the sensitivity of the RI sensors, one may be interested in the limit of detection (LOD). This value is dependent on the standard deviation of the noise over the sensitivty (3σ/S_*λ*_, with σ being smallest measurable value of measurement setup σ = 0.001 nm). For the best performance sensors, the LOD would be in the order of 10^−6^ RIU. A small enough value to measure vitamins such as biotin in the order of 10^−8^ molar [28].

## 7. Conclusions

Various compact RI sensors using silicon sub-wavelength Bragg gratings (SW BG) on SOI platforms have been precisely designed theoretically and demonstrated experimentally. The RI change in the surrounding medium was measured via wavelength shift to evaluate the main wavelength sensitivity (S_*λ*_) as well as the extinction ration (ER) and transmission loss (TL). In order to realize high sensitivity, various structures such as ladder, multi-slot (MS), saw (SAW), complex saw (CSAW), and complex wire (CWIRE) BGs were investigated as well as with and without suspension configuration. It was found that for the non-suspended configuration, sensing using a longer wavelength sidelobe (LSL) is effective, while for the suspended one a shorter one (SSL) is more effective, due to the difference in the mode distributions for the wavelengths relative to the stopband. The adoption of MS-SW BG improved S_*λ*_ due to the enhancement of OSCFs. As for the sensors with suspension, the introduction of more complex structures such as CSAW BG exhibited higher S_*λ*_, but with drawbacks such as difficult fabrication and loss. For the condition of TL smaller than 5 dB, the expected S_*λ*_ is around 900 nm/RIU for CSAW and for SAW BGs with a rather high ER of around 15 dB by Δn = 0.01. As a simpler sensor structure for improved sensitivity, the WG height increase was found to effectively expect an S_*λ*_ of 850 nm/RIU with a WG height of 500 nm.

In the experiments, several methods of EB lithography such as lift-off and inverse-exposure were carried out, and the CSAW BG achieved a high S_*λ*_ of 1100 nm/RIU, using the inverse-exposure method, which was higher than the one expected by the simulation, and this may be attributed to the shape deformation during the fabrication. For the sensor structure that can be fabricated easier, the suspended SAW BG demonstrated a high S_*λ*_ of 913 nm/RIU. The effect of the deformation during the fabrication was discussed.

Though there are several other reports on high wavelength sensitivity RI sensors such as the bimodal WG approach obtaining a high S_*λ*_ of 2270 nm/RIU [33] and the Mach Zehnder structure with 51,495 nm/RIU [35], but these devices are rather long at 100 μm or even longer. As for the high amplitude sensitivity sensors, the MS-SW BG with a large WG height of 950 nm expecting 5000/RIU is also 46 μm [37]. On the other hand, the CSAW and the SAW BG sensors proposed and studied in this paper can exhibit high S_*λ*_ with a very small size as low as 10.2 µm.

Finally, the reported results of both this work and the previous on the BG RI sensors are listed in Table 1. As can be seen with various WG shape modifications, S_*λ*_ values have gradually improved while also reducing their lengths. This work clarified the effectiveness of the compact MS-SW BG RI sensors, and these results indicate that there is still a variety of structures for improving the S_*λ*_ of silicon SW-BG sensors.

## Figures and Tables

**Table 1:** Various BG RI sensors on SOI platform.

Type of BG	Year	H (nm)	S_*λ*_ (nm/RIU)	L (µm)
Middle mesa with top etched grating [24]	2008	1000	33.6	173
Side grating [25]	2009	260	55	15.6
Side grating + PS [23]	2009	N/A	90	13
Slot WG + Side grating [26]	2013	220	340	132
SW with corrugation [27]	2020	220	507	230
Dual-slot + Side gratings [44]	2017	220	661	56.1
Multibox-SW with corrugation+PS [28]	2019	220	579	74
MS-SW [30]	2020	340	730	9.3
MS-SW	* This work	500	850	8.6
Suspended SAW	* This work	220	913	11
Suspended CSAW	* This work	220	1100	10.2

PS—Phase shift. * this work.

**Figure A1:**
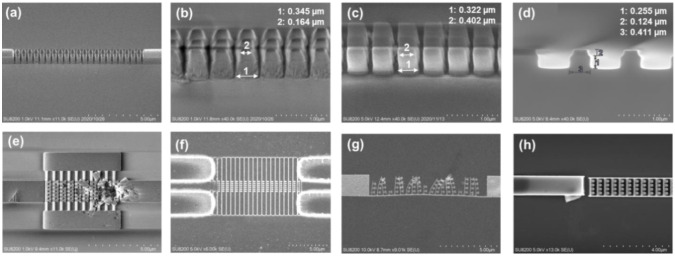
SEM images of (**a**–**d**) MS-SW, (**e**), (**f**) CWIRE, (**g**) CSAW, and (**h**) SAW; MS-SW for WG thickness H = 500 nm in (**a**) zoomed-out and (**b**) zoomed-in views etched with 20 nm Ni mask, and (**c**) zoomed-in view etched with 40 nm Ni mask. (**d**) Etched by inverse exposure method with EBL resist mask thickness H = 520 nm with etching time of 90 s. CWIRE BG fabricated by (**e**) lift-off and (**f**) inverse exposure methods. Destroyed CSAW and SAW BGs due to (**g**) acid cleaning or (**h**) blower usage on the samples after measurements.

## Data Availability

Please email the corresponding author.

## Appendix A. Precautions for Fabrication and Experiment

In this brief section we will note some additional precautions for the fabrication and the experiment. First, when utilizing the lift-off method, a strong etching resistant mask is necessary. For example, nickel (Ni) was used in this work. However, with this selection, undercutting and rounding of the pillar corners may happen. To avoid this the Ni mask can be made thicker. In this work, Ni masks with thicknesses of 20 nm, 40 nm, and 60 nm were considered to fabricate MS-SW BG template patterns with WG height of H = 500 nm. As a result, it was observed that for a thickness larger than 40 nm the defects seem be negligible (shown by Figure A1a–c).

Similarly, top over-etching of the waveguide can occur with the inverse exposure method due to having too thin of an EBL resist protective etching mask. To increase its thickness, the spin rotation speed was lowered for the deposition of EBL resist. Here, 750 rpmi on a 2 cm × 2 cm wafer for 60 s was used for obtaining a thickness of H = 430 nm. The maximal silicon etching depth (while avoiding the side etching issue in this case) was around 255 nm, as seen in Figure A1d, where the black top layer corresponds to the EBL resist. To slightly increase the etching resistance of EBL resist, a post-bake could be used. However, if this is done while there is still some undeveloped residue left, it could affect the following etched patterns by narrowing the etched regions or even leaving them unetched.

Attempts at fabricating CWIRE BG showed the distinct difficulties for both fabrication methods. First, in the lift-off method at the lift-off step, if the deposited metal mask comes off and breaks into small pieces that stick to the substrate, the process may fail, as shown in Figure A1e. To avoid this issue, a pattern can be made such that the metal comes off as one large piece or increasingly small region sizes. That is why CSAW with W_t_ below 1600 nm was unsuccessful. Second, when fabricating the same pattern by inverse exposure, the necessary time for an SiO_2_ BOX layer etching would be longer, as seen in Figure A1f, but this longer etching time would start to etch the regions which were not designed to be suspended.

Now, in the experiments using mediums such as sugar or glycerin mixtures, sample cleaning after measurement is necessary for reproducibility. In this work, piranha acid solution, H_2_O_2_ + H_2_PO_4_ in a 3:1 mixture, was used, and N_2_ gas blower for drying. However, since the patterns under fabrication in this work are rather brittle, breakages were likely to be suffered in these procedures. Therefore, in the later experiments, only water and IPA mediums were used. To make the patterns stronger, thicker supporting walls would be an effective possibly at the cost of lower sensitivity.
